# A Service Evaluation of the Military HeadFIT Initiative: An Implementation Study

**DOI:** 10.3390/ijerph18147375

**Published:** 2021-07-09

**Authors:** Amber McKenzie, Bethany Croak, Laura Rafferty, Neil Greenberg, Sharon A. M. Stevelink

**Affiliations:** 1 King’s Centre for Military Health Research, King’s College London, London SE5 9RJ, UK; amber.1.mckenzie@kcl.ac.uk (A.M.); bethany.croak@kcl.ac.uk (B.C.); laura.rafferty@kcl.ac.uk (L.R.); neil.greenberg@kcl.ac.uk (N.G.); 2 Department of Psychological Medicine, King’s College London, London SE5 8AF, UK

**Keywords:** HeadFIT, mental fitness, military, service evaluation, mental health, wellbeing, initiative, Defence, Civil Service

## Abstract

(1) Background: UK Armed Forces personnel provide first response, support and protection during national and international disasters and conflicts. They thus have a psychologically challenging role which requires them to maintain a good state of mental health and wellbeing. HeadFIT is a preventative initiative developed to help foster mental fitness through various self-help tools and resources online including techniques to de-stress and increase drive. This paper reports on an independent service evaluation of HeadFIT to examine feasibility and acceptability among Ministry of Defence (MOD) personnel. (2) Methods: Qualitative interviews were held with the HeadFIT beneficiaries, including military personnel and civil servants. The beneficiaries provided feedback on HeadFIT through questionnaires and interviews, and website traffic data were also collected. Qualitative data were analysed using framework analysis. (3) Results: Beneficiaries generally reported positive views on the HeadFIT initiative, with most agreeing that the tools could support them to foster their mental fitness. However, concerns were raised around the uptake of HeadFIT and participants suggested methods to improve usability. (4) Conclusions: Several recommendations were made to improve the resources, usability, uptake, and implementation and communication of HeadFIT.

## 1. Introduction

### 1.1. Defence Mental Health 

Military personnel may frequently be exposed to adverse occupational conditions and demands during their service [1] such as operating in stressful environments and responsibility for the lives of others, including during combat, which may contribute to mental health problems [2]. The disciplined occupational environment, and lack of autonomy, within the Armed Forces may also impact the mental health and wellbeing of military personnel [2]. Recent evidence has shown that 21.9% of military personnel who served in the military during the conflicts in Iraq and/or Afghanistan are likely to suffer with a common mental disorder (CMD) [3]. 

### 1.2. Military Mental Health Interventions and Preventions 

In an effort to mitigate any potential psychological impact of military service, the Ministry of Defence (MOD) have launched various initiatives for those displaying symptoms of mental health problems such as the Trauma Risk Management programme (TRiM) [4]. In recent years, increasingly more attention has been paid to proactive mental health interventions, focusing on promoting positive mental wellbeing and preventing mental ill health such as OPSMART [5], SPEAR [6] and Regain [7]. Despite such resources being available, each resource targets a particular military service branch, thereby excluding the Civil Service, and hence limiting the applicability to all Defence personnel. 

### 1.3. The HeadFIT Initiative 

In 2017, the Ministry of Defence and the Royal Foundation announced a collaboration to promote positive mental health and wellbeing and foster mental fitness in current and former Defence personnel. The collaboration’s main initiative is HeadFIT, which is a package that aims to develop and maintain personnel’s mental wellbeing. HeadFIT is intended to provide personnel with the resources and skills to foster their own psychological resilience throughout their Defence career and beyond. HeadFIT seeks to separate ‘mental fitness’ from ‘mental ill health’, which is often associated with stigmatic views within the Armed Forces [8]. Recognising that physical fitness is highly emphasised in the Armed Forces, HeadFIT aims to attach the same level of importance to being mentally fit.

Officially launched in April 2020, HeadFIT is an online mental fitness resource (www.HeadFIT.org access on 6 April 2020) designed for use across the Defence community, including both serving and ex-serving military personnel and Civil Service personnel, unifying one approach to improving individual mental fitness. The HeadFIT tools and resources are categorised into four mental fitness modules: de-stress, drive, confidence, and mood. Each module contains resources informed by the Cognitive Model [9] and the Emotional Regulation Model [10]. For instance, modules include improving body posture, breathing techniques, and self-compassion and acceptance, with the aim to improve mental fitness and reduce the likelihood of mental health issues. 

## 2. Materials and Methods 

### 2.1. Study Population 

Beneficiaries from across the Defence community (Royal Navy, Army, Royal Air Force and MOD Civil Service) were eligible to take part in the HeadFIT service evaluation. All Defence personnel, both military and civilian, were eligible to take part in the evaluation.

### 2.2. Design 

**Figure 1:**
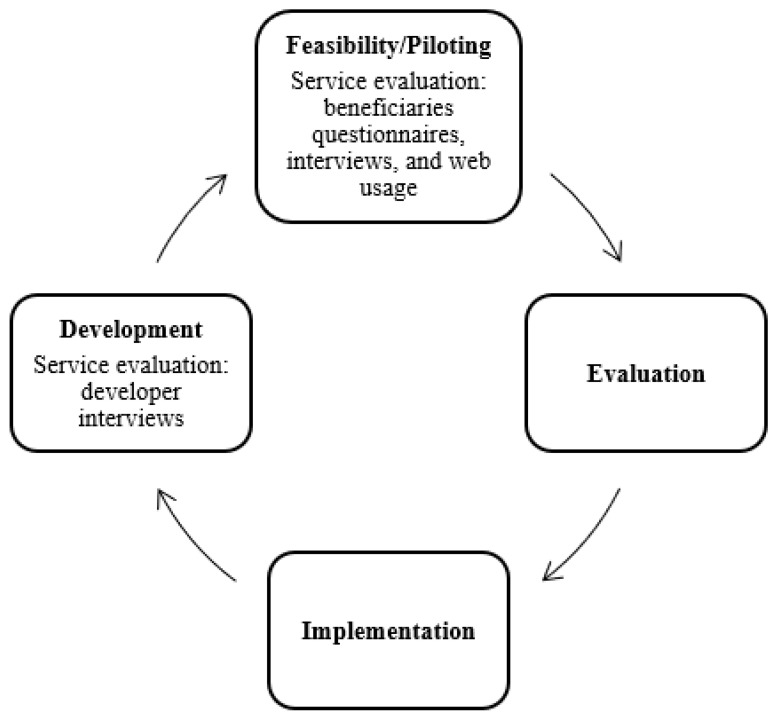
The HeadFIT service evaluation integrated into the MRC Complex Intervention Framework.

The service evaluation applied the Medical Research Council (MRC) ‘Complex Intervention Framework’ [11] (Figure 1), that consists of four main components:

(1) Development of the theory and the intervention;
(2) Feasibility/piloting the intervention with target users;
(3) Evaluation of the effectiveness and changes to the intervention; and
(4) Implementation and dissemination of the intervention.

The service evaluation conducted included a two-pronged evaluation of the development stage and the feasibility/piloting stage.

#### Feasibility/Piloting: Beneficiaries

This paper reports on the main component of the service evaluation which concerned the pilot roll-out of HeadFIT in four military units and among a selection of MOD civil servants working in London. This was evaluated through a qualitative component including interviews with Defence personnel and a quantitative component consisting of a set of three questionnaires completed by beneficiaries. 

### 2.3. Recruitment 

Convenience sampling was used to recruit the beneficiary sample between January and February 2020. We made use of health and wellbeing representatives at each recruitment location including the military units and at MOD Main Building. Each of the five locations were asked to recruit 50–100 potential participants across all ranks and graded through email, word of mouth, the Chain of Command and social media communication platforms. 

### 2.4. Evaluation Procedure and Materials 

#### 2.4.1. Questionnaires 

Beneficiaries were given a baseline questionnaire booklet to complete before watching the 7 min HeadFIT briefing video (BV) which introduced the initiative to beneficiaries. Participants then completed the second questionnaire (AV) before being provided with the HeadFIT URL (www.HeadFIT.org access on 6 April 2020) and encouraged to use the HeadFIT tools. Beneficiaries were asked to complete a follow-up questionnaire (follow-up) 3 months later.

The questionnaires were used to gather beneficiaries’ opinions on ‘mental fitness’, the HeadFIT video and website and their intended use of HeadFIT. Demographic and military/Civil Service characteristics were also collected from the beneficiaries before the HeadFIT briefing video and at follow up. 

#### 2.4.2. Interviews 

Once BV and AV questionnaires were completed, beneficiaries were asked to provide contact details if they were willing to complete an interview to further explore their thoughts on HeadFIT. A semi-structured interview guide was developed to shape the beneficiaries’ interviews focusing on the following core elements: mental fitness, HeadFIT acceptability and impact, HeadFIT usage and communication. The semi-structured interview guides were piloted internally amongst colleagues with no direct affiliation to the evaluation. Beneficiary telephone interviews usually lasted approximately 20–30 min and were audio recorded. Interviews were transcribed by a professional transcription service; all participants were provided with pseudonyms.

### 2.5. Analysis

#### 2.5.1. Questionnaires 

Three separate beneficiary sample groups were created to provide information on the different topics explored.

Sample One (S1) (n = 145) includes all beneficiaries who completed both the BV questionnaire booklet and follow-up questionnaire booklet. This sample was used to explore beneficiaries’ opinions on mental fitness. Data were taken at BV and follow up and were compared to identify any changes to opinion of mental fitness over the course of the HeadFIT pilot. Sample Two (S2) (n = 461) includes beneficiaries who completed the AV questionnaire. This sample was used to explore perceptions on the HeadFIT video and intent to use HeadFIT after the video. Sample Three (S3) (n = 209) includes beneficiaries who completed the follow-up questionnaire. S3 was used to explore perceptions on the HeadFIT website and self-reported website usage. 

#### 2.5.2. Interviews

Interviews were transcribed verbatim and analysed using framework analysis [12]. Framework analysis is appropriate for studies with specific aims, a limited time frame, a pre-designed sample (e.g., professional participants) and an a priori focus (e.g., organisational and integration issues) [12]. The primary researcher (AM) was engaged throughout the entire interview process including creating the interview topic guide, conducting the interviews and analysing qualitative data. Once all interviews were conducted, AM first familiarised themselves with the data from which a coding framework was derived. This coding framework contained sub-themes generated from the topics discussed during each interview. Each segment of the scripts was assigned a code by the primary researcher. Once each interview transcript had been coded, similarities amongst the codes were identified to generate main themes. To increase inter-rater reliability, the identified codes and main themes were discussed and reviewed by the other members of the research team (LR and SAMS) until a consensus was met.

## 3. Results

### 3.1. Questionnaires 

#### 3.1.1. Demographic Information 

Demographic information collected for S1 and S3 is presented below (Table 1). Most of the sample were male and serving in the Army, which is broadly comparable to the demographics of the UK Armed Forces [13,14]. However, most beneficiaries were below 44 years old, with a combined mean age of 35 years (S1 and S3), older than the average age for UK Service personnel. Approximately one in five beneficiaries were in the Civil Service. Whilst data were collected on Civil Service grades, this information has not been included to protect participants’ anonymity due to low numbers.

#### 3.1.2. Opinions on Mental Fitness (S1)

S1 was used to explore differences in opinions on mental fitness before the HeadFIT briefing video (BV) and after beneficiaries had access to the HeadFIT website (follow up).

#### 3.1.3. Definition of Mental Fitness

Approximately one in three beneficiaries defined mental fitness in relation to ‘wellbeing and mental health’, followed by definitions centred around ‘resilience’ and having a ‘positive mental state’. At follow up, fewer beneficiaries described mental fitness as having a ‘positive mental state’, but more in terms of ‘strength’, ‘robustness’ and centred around ‘thought processes impacting behaviour’, whereby the latter two themes newly occurred during follow up (Table 2).

#### 3.1.4. Developing Mental Fitness 

**Figure 2:**
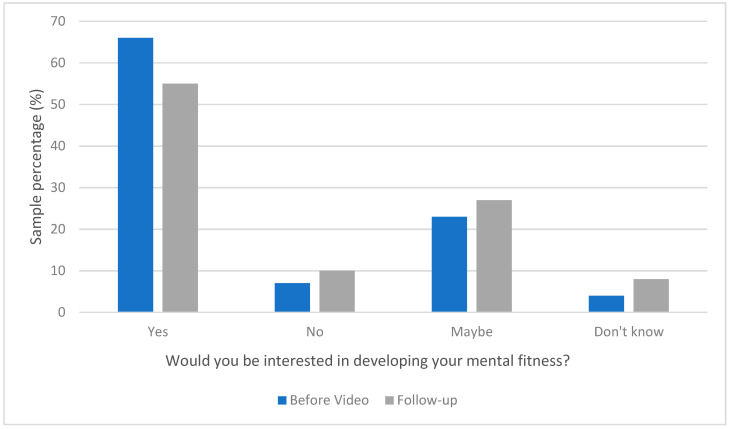
Beneficiaries interest in developing their mental fitness.

Most beneficiaries reported being interested in developing their mental fitness. However, a non-significant deduction in beneficiaries’ interest was revealed when comparing interest before watching the HeadFIT introductory video with follow-up questionnaire data (Figure 2). 

#### 3.1.5. HeadFIT Video Feedback and Intent to Use (S2)

Findings indicated a positive response to the HeadFIT briefing video, with beneficiaries suggesting that the HeadFIT initiative was relevant to them, provided useful information and increased their understanding of mental fitness (Table 3). 

**Figure 3:**
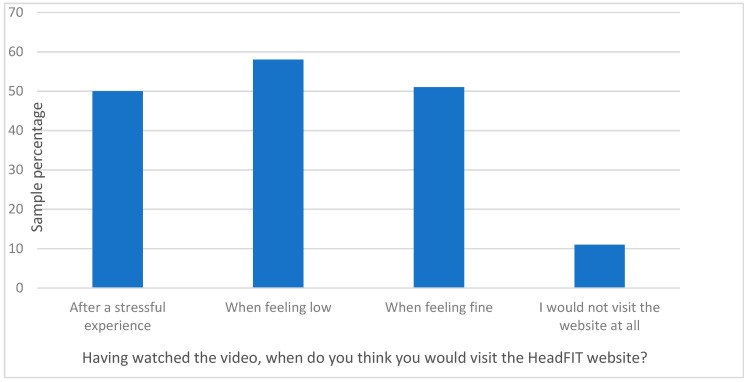
When beneficiaries would visit the HeadFIT website. Note: Beneficiaries were able to select more than one response.

Qualitative feedback from beneficiaries about the HeadFIT briefing video indicated that the psychological models underpinning the HeadFIT initiative, as explained by a counselling psychologist in the video, were the most interesting part. Beneficiaries especially liked that HeadFIT was applicable to all (Armed Forces and Civil Service) and used ‘real’ military personnel in the video. Beneficiaries suggested the briefing video could be improved by adding examples of the HeadFIT tools and possibly some real-life examples/case studies to illustrate the benefits of HeadFIT. Beneficiaries also commented on the lack of representation of Black, Asian and Minority Ethnic personnel, those who are disabled, Civil Service personnel and female personnel in the video. Although HeadFIT was created for Defence personnel to use on a regular basis to improve their mental fitness, around half of the beneficiaries indicated they would only visit HeadFIT after a stressful experience or when feeling low (Figure 3). 

#### 3.1.6. Feedback on the HeadFIT Website and Reported Use of HeadFIT (S3)

##### Reported Use of the Website 

Of the self-reported data about HeadFIT use, 59% (n = 84) reported looking at the HeadFIT website, 29% looking at the tools, with just 8% actually using the HeadFIT tools. When only including participants who had looked at the HeadFIT website (59% of overall sample), 65% reported looking at the HeadFIT tools.

When comparing all the military branches and Civil Service, a higher percentage of Royal Navy beneficiaries reported visiting the HeadFIT website (69%) compared to Army (58%), Royal Air Force (58%) and Civil Service (55%). A higher percentage of Royal Navy also reported looking at the tools (89%) than Army (59%), Royal Air Force (56%) and Civil Service (82%). However, the total number of reported Royal Navy website visitors was relatively low (n = 9) compared to Army (n = 39), Royal Air Force (n = 18), and Civil Service (n = 17). Of those who had reported looking at the website, most reported visiting only once or twice (80%).

Participants were asked to provide free-text responses outlining why they chose to look at the website (or not) and why they chose to use the tools (or not). Of those beneficiaries who reported visiting HeadFIT (59%), the majority (around 7/10) stated they had visited ‘just to have a look’ or to ‘browse’. A small group of participants (around 1/10) reported visiting the website as a result of experiencing poor mental health and a similar number (around 1/10) reported that they had visited the website to see if it was a resource that they might like to recommend to others (employees, friends and family). Most beneficiaries (n = 8) used tools associated with the ‘mood’ module, although total reported use was low. Most beneficiaries reported looking at the HeadFIT tools just to see what was available on the HeadFIT website. 

The free-text responses from those beneficiaries who had not visited the website typically indicated they felt too busy due to work and personal life demands despite wanting to visit the website. Beneficiaries also reported they did not use the tools because their mental health was ‘fine’. 

##### Perceived Ability of HeadFIT to Affect Mental Fitness

Of the beneficiaries who reported visiting the HeadFIT website, 74% felt the HeadFIT tools could improve their mental fitness. Approximately half (52%) thought the HeadFIT initiative had changed the way they thought about mental fitness and how they manage their mental fitness. 

#### 3.1.7. Feedback on the HeadFIT Website 

Most beneficiaries found the website easy to understand, felt that the content was relevant to both their work and personal life and was able to keep their attention (Table 4). 

### 3.2. Interviews

#### Demographics 

A beneficiary sub-group (n = 12) took part in a telephone interview. Interviewees were from the UK Armed Forces and Civil Service: Royal Navy (n = 1); Army (n = 5); Royal Air Force (n = 4); Civil Service (n = 2). Eight were male, four were female, and their mean age was 37. Interviewees ranks consisted of: Senior Non-Commissioned Officer (n = 4); Junior Non-Commissioned Officer (n = 1); Junior Commissioned Officer (n = 2); Senior Commissioned Officer (n = 2); and other ranks (n = 1). Both Civil Service beneficiaries were from a non-skill zone grade. 

**Figure 4:**
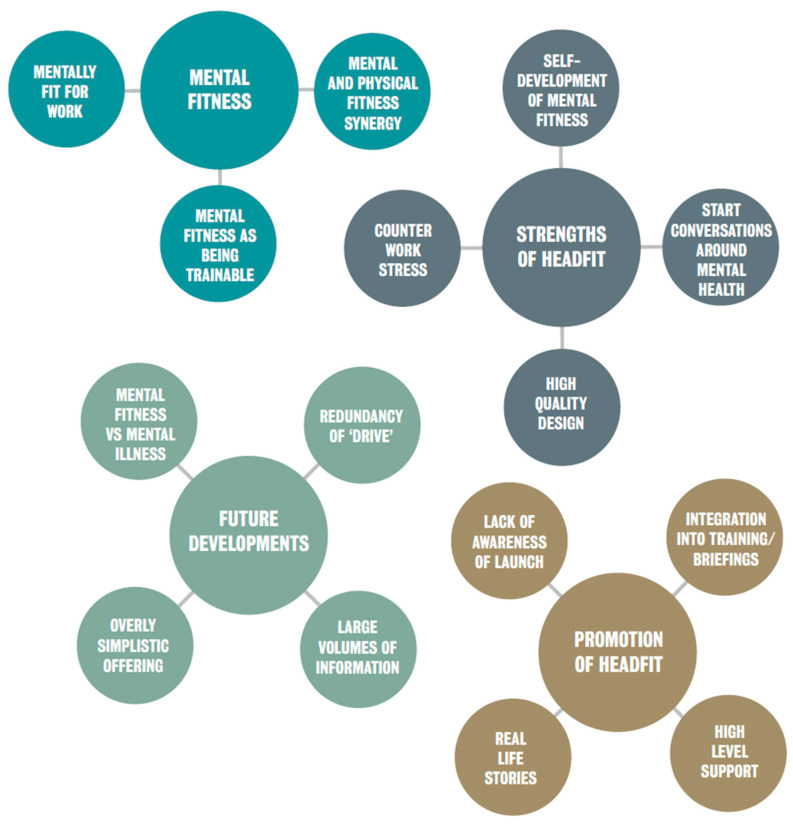
Themes and sub-themes from beneficiary interviews.

Four main themes, along with associated sub-themes, were identified from the interviews with beneficiaries: (1) Mental fitness; (2) Strengths of HeadFIT, (3) Future developments; (4) Promotion of HeadFIT (Figure 4).

(a) Mental fitness

Mental and physical fitness synergy 

Without prompt from the interviewer, most beneficiaries related mental fitness to physical fitness, highlighting the two as equally important. “*Personally, I think being mentally fit is just as important as being physically fit. I think you need to have a balance of both.”* [R9]

Mental fitness as being trainable 

Interviewees spoke positively about the way in which HeadFIT provides exercises to actively improve mental fitness rather than only providing education on mental fitness. *“And then it’s talking about breathing exercises and specific things you can actually physically do to improve your mental fitness. The point is they’re all doing words, they’re all verbs as in like they’re [tools] all things that you can actually do not think about they’re things that you can actually go and practice.”* [R1]

(b) Strengths of HeadFIT

Practical tools to counter work stress

A common theme reported was that HeadFIT could help to alleviate work-related stress. Especially as military work is stressful, and personnel are often busy and overworked, highlighting the benefits of the ‘de-stress’ components. All interviewees felt that the HeadFIT exercises could help Defence personnel perform better and would be helpful to personnel if they were ‘a bit stressed’ or in a ‘bad mood’: *“I think stress is probably the one that I could identify with or as in I think that the majority of people, I see in work who might need this sort of tool.”* [R1] 

Starting conversations around mental health 

Interviewees felt that HeadFIT could have a positive impact upon military and Civil Service personnel by stimulating conversations surrounding mental fitness and mental health. Some (n = 4) felt that HeadFIT supported individuals in becoming aware of other’s mental health and how to navigate mental health at work. Interviewees also reported speaking to others about HeadFIT and mental fitness, with some recommending HeadFIT to colleagues whose mental health had been negatively impacted: *“We have to look ourselves and others to make sure that they’re OK not only in body but in mind as well…… I’ve recommended it [HeadFIT] to line managers to push it out there towards the other users on the section. So they are aware of it.”* [R2] 

However, it is important to note that some interviewees only reported speaking to others about HeadFIT directly after the HeadFIT briefing and not since the briefing, suggesting that HeadFIT might only encourage conversations about mental health in the short term.

(c) Future developments

Mental fitness vs. mental illness

An important area for development was around the concept of mental fitness. Whilst many commented that HeadFIT had altered mental fitness perceptions, a proportion still felt that work was needed to overcome the stigma associated with mental wellbeing. It was highlighted that further work is needed to differentiate mental fitness from mental illness. Most beneficiaries who visited the website commented they did so ‘just to have a look’, with most stating that they felt ‘fine’ and had no reason to use the tools, emphasising their view of HeadFIT as a resource to use when your mental health has been negatively impacted rather than regular use to upkeep mental fitness: *“I felt alright these past [weeks], since the meeting so I’ve not felt that I’ve had to go out and look for something to improve my mental fitness. I think my mental fitness is OK at the minute, so I don’t need to develop it.”* [R8]

Redundancy of ‘Drive’ tools

Some beneficiaries felt the ‘Drive’ section demonstrated a lack of understanding about the Defence community, commenting that military personnel do not have a problem with drive, and they are in fact over driven: *“Most people in the military are quite driven usually I think so the drive aspect I think usually we don’t have a problem seeing people lacking drive. It’s usually that they drive themselves too hard and they just don’t relax and it’s all a bit too serious and so therefore the destressing tools.”* [R1] 

Personalisation

Some beneficiaries suggested that personalising the HeadFIT experience may be beneficial, for example adding the ability to log in and track individual progress through the content: “It’s perhaps a little bit more confusing than it needs to be… I think there’s going to be information there that people aren’t looking at. I also think there is probably too many words on it. I think there’s a lot of reading for each section.” [R3]

(d) Promotion of HeadFIT

Lack of communication around HeadFIT

All beneficiaries agreed that communication would be important for the success of HeadFIT. However, most were not aware that HeadFIT had been officially rolled out. Concerns were raised that the COVID pandemic impacted awareness due to working alterations: “There were other pieces coming out as well and then obviously with the Covid-19 and stuff like that going on these have been things that I think a lot of things have got lost in the mayhem haven’t they.” [R2] 

Integration into training/briefings

Several beneficiaries suggested that HeadFIT should be integrated into routine training and face-to-face briefings, specifically phase one training which recruits receive to instil the importance of mental fitness at the beginning of service. They felt that doing this would help to support a shift in thinking around mental health and mental fitness and contribute to reducing mental health stigma: “No one is training a 17-year-old who is going through army training on confidence, mood and drive.” [R1]

High-level support

Most beneficiaries felt that HeadFIT needed to be championed from the top-down to improve awareness and encourage use. Interviewees felt that having higher-ranking personnel use HeadFIT would begin to tackle mental health stigma and encourage habitual use of HeadFIT: “Actually if it’s rolled out I would really like my bosses to be talking about it and be part of the conversation. I think it would be really important now.” [R7] 

Real-life stories 

To increase HeadFIT engagement, several beneficiaries suggested incorporating personnel who used the tools and website as real-life examples of how HeadFIT can improve mental fitness. For instance, telling their story and describing how HeadFIT had impacted their personal and work life in order to encourage others to use the tools: “If some people are using the tools and are prepared to speak about using those tools to others within their work context then that does more than anything else with that personal recommendation, I use this and I would recommend it. That’s what gets more take up than absolutely anything else.” [R5]

## 4. Discussion 

Mental health initiatives have been identified as important given the psychologically challenging duties that military personnel routinely undertake [2]. Literature has highlighted a lack of research into the impact of military interventions and recommendations to improve interventions based on empirical findings [15,16]. The current service evaluation aimed to assess the feasibility and acceptability of the HeadFIT initiative to inform evidence-based recommendations for further improvements.

The HeadFIT initiative aims to separate the terminology of mental fitness from that of mental ill health, encouraging beneficiaries to maintain their mental fitness in a similar way to their physical fitness. There is very limited research which distinguishes mental fitness from mental health. Nevertheless, the interventions to improve mental fitness or mental ill health differ drastically in their practices. It can be described as a positive term void of the nuance of illness as implied by the term ‘mental health’ [17]. However, the evaluation showed that this aim of separating both terminology within HeadFIT was not met. Although beneficiaries believed that HeadFIT provided them with practical tools to improve mental fitness and deal with the challenging work circumstances, data suggested that the majority only intended to utilise the HeadFIT resources when experiencing mental ill health and not to promote mental wellbeing or fitness. Therefore, it is recommended that a clearer message distinguishing when HeadFIT should be used, and differences between mental fitness and mental health would increase beneficiaries’ awareness of when to utilise HeadFIT. More broadly, there is room for future research to provide a clear distinction between both concepts which would further assist developing mental fitness initiatives. 

The findings from the service evaluation emphasised the importance of a thorough communications and implementation strategy to ensure widespread and sustainable uptake of HeadFIT. Previous research supports the notion of communication as a crucial component to intervention uptake [18]. Research into barriers and facilitators of uptake of staff health and wellbeing services in the NHS found that staff believed word of mouth and face-to-face communication within the work force would be a more effective method of communication than email [19]. Encouragement by senior leadership, testimonials from people who had used HeadFIT (champions), and integration into routine training were all purported to be a way forward to improve uptake. Research shows that employing wellbeing champions and senior management encouraging wellbeing initiative leads to improved mental health for not only the wider community but for the champion themselves [20]. Interestingly, our findings suggested that increasing awareness and encouraging uptake of HeadFIT via email communications was expected to be unsuccessful, especially for junior ranks. This may be explained by the impact of the COVID pandemic on HeadFIT communication. A reduction in face-to-face military briefings and an increase in remote communication may have distracted attention from the HeadFIT. Therefore, we would recommend that an additional evaluation of the HeadFIT implementation and communication strategy should be conducted once normal Defence working resumes as this would provide a more accurate evaluation of its efficacy. 

Beneficiaries also reported a lack of ability to personalise HeadFIT, including the inability to create a HeadFIT account allowing users to track and monitor personal usage may have impacted usage. Similar findings were echoed in a study reviewing a mental health self-help app that found lack of personalisation impacted engagement with the app [21]. Future iterations of HeadFIT should incorporate the ability to personalise resources and provide an option to create an account, track usage, receive updates on new resources and reminders. Further, creating a HeadFIT mobile app could enable personalisation and facilitate progress tracking. 

### 4.1. Strengths

The aim of the service evaluation was to explore the acceptability and feasibility of the HeadFIT initiative among members of the UK Defence community. Research has shown that negative attitudes towards mental health treatment within military populations can act as a barrier to care [8]. It was hoped that the evaluation would foster confidence in the initiative if there was evidence of acceptability and feasibility, and if evidence was lacking, help refine the HeadFIT initiative.

### 4.2. Limitations 

The evaluation was initially designed to match the beneficiaries’ demographic information from the follow-up responses to their BV and AV responses to enable a direct comparison of the beneficiaries’ opinions before HeadFIT compared to after the initiative. However, this was not possible. At times, questionnaire responses proved difficult to match for several reasons including demographic information partially completed or illegible, and an unforeseen change to an online survey format as a result of the COVID restrictions instead of the planned follow-up base visits. This resulted in the need to create three beneficiary sample groups rather than one overall beneficiary sample. 

COVID and the move to online questionnaires had additional implications including a lower response rate for the follow up and impacted recruitment for beneficiary interviews, resulting in a smaller sample than anticipated. Further, several military units involved in the evaluation were deployed to support the COVID response. These obstacles may have impacted on the findings’ validity. The service evaluation also used a convenience sampling strategy due to practical and feasibility purposes. As such, our sample is not representative of the whole Defence community. 

Beneficiaries were asked to provide contact details if they would be willing to take part in the interviews. Sampling in this way may have created a biased view in the interviews in that only those who viewed the initiative in a positive light may have been willing to take part. In the future, it might be pertinent to interview individuals from distinct groups: those who reported they had used HeadFIT and those who reported they had not use it.

## 5. Conclusions

Overall, the service evaluation found HeadFIT was well received by the target beneficiaries, with most agreeing that it provided a set of tools to support individuals in their development of mental fitness. Concerns were raised surrounding the widespread uptake of HeadFIT and many envisioned challenges in ensuring beneficiaries regularly use the tools to improve their mental fitness rather than accessing HeadFIT only when experiencing mental ill health. Distinctions between mental ill health and mental fitness were often blurred for beneficiaries. It seems likely that this mismatch may impact the acceptability and use of HeadFIT. However, the impact from COVID pandemic on the service evaluation must be considered. The HeadFIT initiative would benefit from future research into the effectiveness of the service for improving mental fitness for Defence personnel. 

## Figures and Tables

**Table 1:** Description of Beneficiaries’ Demographics and Occupational Factors.

Variables	S1	S3
	n (%)	n (%)
Total Number of Beneficiaries	145	209
Age		
<24	21 (15%)	47 (23%)
25–34	48 (33%)	69 (34%)
35–44	40 (28%)	49 (24%)
45–54	23 (16%)	27 (13%)
55+	12 (8%)	13 (6%)
Mean (SD) ^a^	36 (10.9)	34 (10.8)
Gender		
Male	111 (77%)	160 (78%)
Female	34 (23%)	45 (22%)
Service Branch		
Royal Navy	15 (10%)	17 (8%)
Army	62 (43%)	116 (57%)
Royal Air Force	36 (25%)	38 (18%)
Civil Service	31 (22%)	34 (17%)
Military Service Rank		
Senior Commissioned Officer	9 (8%)	9 (5%)
Junior Commissioned Officer	22 (19%)	28 (17%)
Senior Non-Commissioned Officer	31 (27%)	39 (23%)
Junior Non-Commissioned Officer	26 (23%)	40 (23%)
Other Ranks	24 (23%)	55 (32%)
Military Role		
Combat and Combat Support Role	22 (21%)	26 (15%)
Combat Support Role	84 (79%)	140 (85%)

Note. The N participant number for each sub-section may not equate to the total N participant number for each sample due to missing data. ^a^ SD = standard deviation.

**Table 2:** Beneficiaries’ Definitions of Mental Fitness Comparing BV to Follow Up.

Definitions of Mental Fitness	BV (%)	Follow Up (%)
Wellbeing and mental health	☒(34%)	☒(35%)
Resilience	☒(15%)	☒(17%)
Positive mental state	☒(15%)	☒(7%)
Stress management	☒(9%)	☒(5%)
Coping	☒(6%)	☒(1%)
Strength	☒(6%)	☒(9%)
Robust mental state	☐(-)	☒(5%)
Thought processes impacting behaviour	☐(-)	☒(4%)
Other	☒(10%)	☒(17%)

**Table 3:** Beneficiaries Feedback on the HeadFIT Briefing Video.

Feedback Statements	Agree	Disagree
	n (%)	n (%)
I think the HeadFIT tools could be useful to me	391 (87%)	60 (13%)
I know where to find the HeadFIT tools	376 (84%)	51(16%)
I know which HeadFIT tools are available to me	306 (69%)	136 (31%)
The video has increased my understanding of what mental fitness means	350 (79%)	96 (21%)
I now know more about how to manage my mental fitness	332 (74%)	112 (26%)
The video was the right length	434 (96%)	18 (4%)
The video was relevant to me	372 (83%)	79 (17%)
The video used good examples	368 (83%)	75 (17%)
The video provided me with important information	378 (84%)	71 (16%)

Note: Beneficiary responses may not correspond to the total number of beneficiaries in S2 due to missing data.

**Table 4:** Beneficiaries’ HeadFIT Website Feedback.

Feedback Statements	Agree	Disagree
	n (%)	n (%)
I found the HeadFIT website too complicated	8 (10%)	68 (90%)
I thought the HeadFIT website was easy to understand	71 (94%)	5 (6%)
I found the tools in the HeadFIT website were put together well	59 (93%)	5 (7%)
I found the HeadFIT website difficult/awkward to use	8 (11%)	67 (89%)
I had to take some time to learn how to use the HeadFIT website before I could use it properly	23 (32%)	50 (68%)
The HeadFIT website content was relevant to my work	64 (86%)	10 (14%)
The HeadFIT website content was relevant to my personal life	66 (91%)	7 (9%)
I found the HeadFIT website kept my attention	59 (79%)	16 (21%)

## Data Availability

The data presented in this study are available on request from the corresponding author.
